# Ten-Year Legacy Effects of Three Eight-Month Exercise Training Programs on Cardiometabolic Health Parameters

**DOI:** 10.3389/fphys.2019.00452

**Published:** 2019-04-16

**Authors:** Johanna L. Johnson, Cris A. Slentz, Leanna M. Ross, Kim M. Huffman, William E. Kraus

**Affiliations:** ^1^Duke Molecular Physiology Institute, Duke University Medical Center, Durham, NC, United States; ^2^Division of Cardiology and Urbaniak Sports Sciences Institute, Duke University Medical Center, Durham, NC, United States

**Keywords:** cardiovascular health, cardiorespiratory fitness, risk factors, intervention, longitudinal follow-up, exercise intensity, exercise amount

## Abstract

**Background:** STRRIDE (Studies Targeting Risk Reduction Interventions through Defined Exercise) was an eight-month exercise study conducted from 1998–2003. Subjects were randomized to control or one of three exercise groups differing in intensity and amount. To determine if there were legacy effects, we invited 161 individuals who completed the intervention phase to return for a 10-year Reunion study.

**Methods:** Subjects completed medical history and physical activity questionnaires. Height, body weight, blood pressure, waist circumference, and peak VO_2_ were measured. Fasting blood samples were analyzed for glucose, insulin and lipids. Of 161 original subjects, 153 were within 10 years of STRRIDE completion. Of these, 28 were lost to follow-up and 21 declined to participate in the Reunion study. Overall, 104 subjects (83% eligible) participated. Change over time was computed as the 10-year Reunion value minus the pre-intervention value. Significant within group changes were calculated using two-tailed *t*-tests. ANCOVA determined differences among groups with pre-intervention values as covariates. Bonferroni corrections were applied to account for multiple comparisons.

**Results:** Ten years after completing STRRIDE, there were a number of group-specific health and fitness legacy effects. Original participation in either the moderate intensity exercise or control group resulted in a 10.5% decrease in peak VO_2_ over the ensuing 10 years. Conversely, both vigorous intensity groups experienced only a 4.7% decrement in cardiorespiratory fitness over that time period. As compared to controls, all three exercise groups experienced smaller increases in waist circumference. Those who participated in moderate intensity exercise experienced the greatest 10-year reduction in fasting insulin. Compared to all other groups, the moderate intensity subjects had greater reductions in mean arterial pressure at the Reunion timepoint.

**Summary:** Ten years after completing a randomized eight-month exercise training intervention, previously sedentary individuals exhibited group-specific differences consistent with an intervention-based legacy effect on cardiorespiratory fitness and cardiometabolic parameters. These findings highlight the critical need to better understand the sustained legacy health effects of exercise training interventions.

## Introduction

According to the 2008 United States Physical Activity Guidelines Advisory Committee Report, “Very strong scientific evidence based on a wide range of well-conducted studies shows that physically active people have higher levels of health-related fitness, a lower risk profile for developing a number of disabling medical conditions, and lower rates of various chronic diseases than do people who are inactive” (Physical Activity Guidelines Advisory Committee, 2008). In previously sedentary subjects, aerobic exercise interventions lead to improvements in cardiorespiratory fitness and cardiometabolic disease risk factors. However, whether a period of supervised exercise training of modest duration (e.g., 8-month) can result in persistent long-term (legacy) benefits is unknown.

In medicine, a legacy effect describes the sustained benefit of a treatment long after cessation of the treatment which was given in the early phase of disease (Coppo, 2013). The legacy effect concept was first discussed in the medical literature by Holman et al. (2008) regarding 10-year post-trial follow-up of the United Kingdom Prospective Diabetes Study. The original study, a randomized controlled trial, investigated early and strict control of diabetes in over 5,000 patients with newly diagnosed type 2 diabetes mellitus. Although between-group differences in glycated hemoglobin were lost within one year of treatment cessation, over 66,000 person-years of follow-up revealed significant, persistent cardiovascular benefits of intensive blood glucose control therapy (sulfonylurea-insulin or metformin) on micro- and macrovascular complications compared to patients randomized to the conventional therapy group (Holman et al., 2008). Additional results supporting potential legacy health effects have been documented from long-term follow-up of lipid-lowering statin trials, including reduced cardiovascular events and all-cause mortality, and increased quality-of-life compared to controls (Sever et al., 2011; Kashef and Giugliano, 2016).

Furthermore, in three major diabetes prevention follow-up studies, intense lifestyle interventions — involving diet, exercise and weight loss goals — resulted in persistent beneficial effects up to 10 years following trial cessation. The Finnish Diabetes Prevention Study reported both improved lifestyle and decreased diabetes risk over 13 years compared to controls (Lindstrom et al., 2013). In the Chinese Da Qing Diabetes Prevention Study, lifestyle interventions (exercise-only, diet-only, or exercise plus diet) conducted over 6 years prevented or delayed diabetes for up to 14 years after the active intervention compared to participants in the control group (Li et al., 2008). In the Da Qing study, there was a reduced incidence of cardiovascular and all-cause mortality and diabetes 23 years after the intervention (Li et al., 2014). In the 10-year follow-up of the Diabetes Prevention Program, a reduced incidence of diabetes persisted in the lifestyle group for 10 years following the end of the study (Diabetes Prevention Program Research Group, 2009). Thus, long-term follow-up after the end of lifestyle trials provides substantial evidence for legacy effects resulting from even relatively short-term interventions. These long-term benefits reported from the diabetes prevention studies could be the result of the sustained effects of the single intensive intervention itself, or that there may be long-term effects on healthy lifestyle behaviors, or both.

Despite these encouraging reports, there remains little information about the legacy effects of exercise-based programs on long-term cardiorespiratory fitness and metabolic health benefits, and whether responses differ by the intensity or total energy expenditure of the exercise training program. In STRRIDE (Studies of Targeted Risk Reduction Interventions through Defined Exercise), an 8-month randomized, controlled exercise study, the numerous metabolic health and cardiorespiratory benefits were often specific to the exercise intervention group. These intervention groups exposed subjects to different amounts and intensities of exercise training, ranging from fairly substantial to more modest amounts and intensities. In light of the numerous studies of intensive lifestyle interventions (i.e., diet and exercise) and the findings of long-term legacy benefits, we invited our STRRIDE subjects who completed the original study back for testing 10 years later to determine if there were any legacy benefits, and if there were, whether they were specific to the exercise training group to which they were originally assigned.

## Materials and Methods

The original STRRIDE study (1999–2003) enrolled previously sedentary, overweight or obese men and women aged 40–65 years with mild to moderate dyslipidemia [classified by low-density lipoprotein cholesterol (LDL-C): 130–190 mg/dL or high-density lipoprotein cholesterol (HDL-C): ≤ 40 mg/dL for men and ≤ 45 mg/dL for women] (Kraus et al., 2001). Subjects were randomized into one of four groups: (1) inactive control; (2) low amount/moderate intensity exercise: 14 kcal/kg of body weight/week (KKW) at 40–55% peak oxygen consumption (VO_2_); (3) low amount/vigorous intensity exercise: 14 KKW at 65–80% peak VO_2_; (4) high amount/vigorous intensity: 23 KKW at 65–80% peak VO_2_. Subjects originally randomized to the control group were offered the opportunity to be randomized to one of the three exercise interventions at the end of their control period. For subjects who successfully completed the subsequent exercise training, data collected during their control period was not used in analysis.

All 161 individuals from the Duke study site who completed the original STRRIDE study were invited to return for a “Reunion” study. For practical and geographical reasons, subjects who participated at the East Carolina University study site were not invited to the Duke Reunion study. Subjects were invited approximately 10 years following their study completion. The Reunion study was approved by the Duke University Institutional Review Board. Subjects signed written informed consent.

For the 10-year Reunion study, subjects completed medical history and physical activity questionnaires. Expanding upon the Paffenbarger Physical Activity Questionnaire (Paffenbarger et al., 1978), subjects completed a 23-item assessment of frequency, duration, mode, and intensity of recent physical activity participation. Subjects also underwent height, body mass, resting blood pressure, minimal waist circumference (Willis et al., 2007), and cardiorespiratory fitness assessments. Subjects were asked to provide a fasting blood sample.

Fasting blood samples were obtained from 103 of the Reunion subjects. Plasma was analyzed for HDL-C, triglycerides and glucose with a Beckman-Coulter CxC600 clinical analyzer (Brea, CA, United States). In the original STRRIDE study, plasma glucose was determined via a YSI analyzer (Yellow Springs, OH, United States), and HDL-C as well as triglycerides were determined via nuclear magnetic resonance spectroscopy (LipoScience, Raleigh, NC, United States). Reunion plasma insulins were measured by electrochemiluminescent plate assay (Meso Scale Discovery; Gaithersburg, MD, United States), whereas plasma insulin in the original STRRIDE study were determined via immunoassay (Access Immunoassay System, Beckman Coulter, Fullerton, CA, United States). We reasoned that although different assays were used before and after the 10-year interim, the calculated change scores should not be biased by group assignments, permitting an assessment of differences among the various exercise intensity and amount exposures over time (Marcovina et al., 2007; Staten et al., 2010); However, statistically significant change scores within a single group should be interpreted with caution—particularly with the fasting glucose measure — due to differences in assay parameters that may have been used in each of the original STRRIDE and Reunion studies.

On a separate day, 89 subjects (of the 104) completed graded maximal treadmill exercise testing with 12-lead electrocardiography and peak oxygen consumption (peak VO_2_) was measured via gas exchange (TrueMax Parvomedics; Provo, UT, United States). As an additional marker of cardiorespiratory fitness, we assessed submaximal exercise capacity by determining VO_2_ at anaerobic threshold using the V-slope method (Beaver et al., 1986). A plot of 15-s averages of VO_2_ versus VCO_2_ from each exercise test was given to two separate readers in order to determine the VO_2_ (mL/min) at which the anaerobic threshold occurred. If the two reads were within 150 mL of each other, the value for VO_2_ at anaerobic threshold was considered to be the average of the two chosen values. If the values differed by more than 150 mL, the plot was given to a third reader to confirm one of the two reads. If the third reader was not in agreement with either of the first two readers, the VO_2_ at anaerobic threshold for that subject’s test was not included in the analysis. The result was 52 subjects with complete anaerobic threshold data.

Means and standard deviations were determined for each of the variables collected at the 10-year Reunion visit. Absolute change scores were calculated as the difference between the 10-year Reunion value minus the pre-training intervention value. We also calculated percent change as the absolute change score divided by the pre-training value. This permitted us to compare the combined effects of randomization, participation and differential post-training behavior among the groups for the entire intervention and follow-up periods. To determine statistical significance for within group effects, two-tailed *t*-tests were used; a *p*-value of 0.05 was used to indicate statistical significance. For among-group effects, an analysis of variance (ANCOVA) was used with baseline values considered covariates; to correct for multiple comparisons, an overall *p*-value of 0.05 with Bonferroni *post hoc* analyses were performed. With four groups, there are six possible pairwise comparisons; therefore, alpha level for significance was set at *p* ≤ 0.0083 (0.05/6 comparisons) for between groups comparisons. To address potential differences in change in fasting plasma glucose based upon medication usage that might affect glucose concentrations (e.g., beta-blockers, thiazides, dedicated glucose lowering medication), we performed an additional ANOVA that separated subjects into two groups (glucose-affecting medication use yes/no). When assessing change in blood pressure among the original intervention groups, blood pressure medication usage (yes/no) was used as a covariate in our ANCOVA model. In addition, we calculated Pearson correlation coefficients to assess the relationship between self-reported exercise frequency over the last three months (taken at the STRRIDE Reunion timepoint) and change in cardiometabolic health variables. All statistics were conducted using STATVIEW (SAS, Cary, NC, United States).

## Results

At the Duke site, there were 161 intervention completers from the original STRRIDE study; of these, 153 were within the 10-year (± six-month) window. Of the 153, 28 were lost to follow-up (7 moved, 15 unable to be contacted, 5 deceased, 1 was in another exercise study) and 21 declined. Therefore, out of the possible 125 subjects, 104 subjects came in for testing — an 83.2% return success rate. Of the 104 subjects, 13 elected not to do the maximal treadmill test and in one subject we were not able to obtain a blood sample. Of the 104 subjects (mean age 63.0 ± 6.2 years; 77.9% Caucasian), 52 were men and 52 were women; this compares to 101 men and 87 women who completed the original STRRIDE study at Duke. Over the ensuing 10 years, on average, the BMI was not changed: 29.9 kg/m^2^ versus 29.9 kg/m^2^. Table 1 contains baseline (pre-randomization) metabolic characteristics for the original STRRIDE study data, as well as the data for these same variables obtained from the Reunion study. In addition, the statistical significance of the change within a specific group over this time period is indicated for each variable.

**Table 1:** STRRIDE baseline (pre-randomization) and STRRIDE Reunion (10 years and 8 months later) values for cardiometabolic health variables.

	Control		Low Amount / Moderate Intensity		Low Amount / Vigorous Intensity		High Amount / Vigorous Intensity	
	(*n* = 10)		(*n* = 28)		(*n* = 35)		(*n* = 31)	
Variable	Baseline	Reunion	Baseline	Reunion	Baseline	Reunion	Baseline	Reunion
Body Mass (kg)	84.8 (9.5)	88.1 (8.3)	85.0 (14.1)	84.9 (14.8)	86.4 (13.1)	85.0 (14.9)	84.4 (13.1)	84.0 (13.7)
Minimal Waist Circumference (cm)	92.2 (7.2)	97.2 (6.8)*	95.0 (10.9)	95.4 (10.8)	94.0 (9.2)	93.9 (9.9)	94.3 (11.7)	95.6 (11.9)
Systolic Blood Pressure (mmHg)	125.0 (15.8)	125.2 (12.0)	127.6 (12.4)	118.8 (10.4)*	122.8 (10.4)	124.7 (15.0)	126.6 (9.6)	123.0 (14.1)
Diastolic Blood Pressure (mmHg)	80.2 (8.1)	77.7 (10.6)	80.0 (5.6)	72.9 (8.9)***	79.2 (5.8)	77.1 (8.3)#	80.4 (6.2)	76.9 (8.5)*
MAP (mmHg)	95.1 (9.9)	93.5 (9.7)	95.9 (7.2)	88.2 (8.8)***	93.7 (6.2)	93.0 (8.8)	95.7 (6.4)	92.3 (9.2)*
HDL (mg/dL)	47.7 (14.4)	44.4 (10.3)	46.9 (14.5)	45.6 (10.5)	46.8 (12.4)	48.7 (20.4)	46.1 (16.6)	47.6 (15.3)
Triglycerides (mg/dL)	152.7 (66.3)	144.7 (69.9)	153.9 (90.0)	111.6 (57.8)*	131.1 (57.7)	107.6 (45.9)*	137.2 (62.9)	113.4 (85.2)#
Fasting Glucose (mg/dL)	85.5 (8.5)	99.3 (6.4)***	92.8 (8.8)	107.7 (15.7)***	94.1 (9.2)	104.7 (16.8)***	91.5 (8.7)	106.8 (14.6)***
Fasting Insulin (uU/mL)	6.6 (3.2)	7.5 (3.2)	10.3 (9.2)	5.9 (3.5)*	8.7 (6.4)	6.2 (4.5)*	7.2 (3.8)	6.8 (3.9)
Peak VO_2_ (ml/kg/min)	26.1 (6.1)	22.9 (4.5)*	28.5 (6.5)	25.5 (6.6)***	29.4 (6.0)	28.0 (6.3)#	29.3 (5.4)	27.6 (5.8)#
Time to Exhaustion (sec)	728.9 (231.9)	682.9 (186.3)*	785.4 (247.6)	749.6 (242.1)	778.0 (183.9)	805.7 (204.9)	796.1 (198.0)	754.5 (191.9)
ATP III Metabolic Syndrome score	1.9 (.88)	2.7 (.82)*	1.9 (.99)	2.0 (1.2)	1.6 (.98)	2.3 (1.3)*	1.8 (1.2)	2.4 (1.5)*
Metabolic Syndrome Z Score	-2.3 (1.7)	-0.350 (2.7)*	-1.3 (2.7)	-1.7 (2.6)	-2.1 (2.1)	-1.2 (4.2)	-1.8 (2.9)	-0.805 (4.3)


*Values are means (SD). ANOVA revealed no significant baseline differences between groups, except for fasting glucose, which was significantly or nearly significantly lower in the control group compared to the other three groups. MAP, Mean Arterial Pressure; HDL, High Density Lipoprotein; Peak VO_*2*_, peak oxygen consumption; ATP III, National Cholesterol Education Program’s Adult Treatment Panel III criteria. ^#^*P* < 0.10, ^∗^*P* < 0.05, ^∗∗∗^*P* < 0.001.*

### Cardiorespiratory Fitness

**FIGURE 1:**
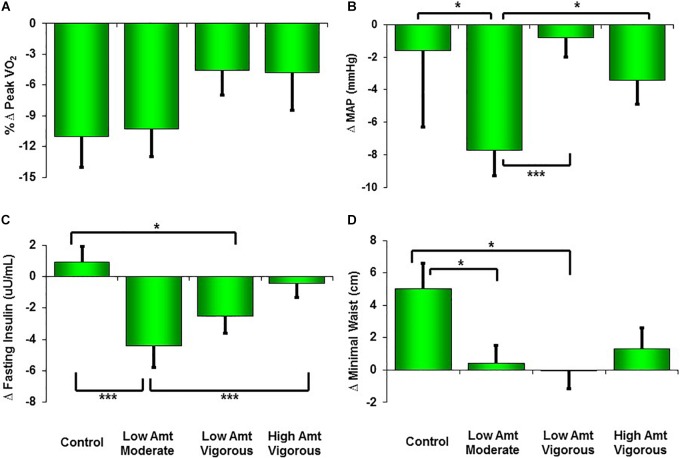
Changes from pre-STRRIDE to STRRIDE Reunion (10 years and 8 months later) for peak VO_2_ **(A)**, mean arterial blood pressure (MAP; **B**), fasting insulin **(C)**, and minimal waist circumference **(D)**. Amt = Amount; ^∗∗∗^ represents *P* < 0.0083, which indicates significant difference among groups after correction for multiple comparisons (Bonferroni); ^∗^ represents *P* < 0.05 (not significant difference among groups after Bonferroni corrections).

**FIGURE 2:**
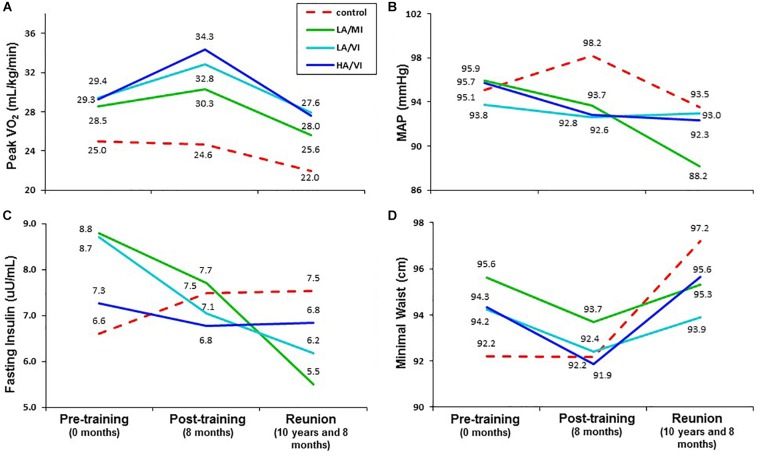
Mean values for pre-training ( 0 months), post-training (8 months) and STRRIDE Reunion (10 and 8 months) for peak VO_2_ **(A)**, mean arterial blood pressure (MAP; **B**), fasting insulin **(C)**, and minimal waist circumference **(D)**. Dotted red line represents control group; solid green line represents low amount/moderate intensity group (LA/MI); solid light blue line represents the low amount/vigorous intensity group (LA/VI); and solid dark blue line represents the high amount/vigorous intensity group (HA/VI).

As expected, after more than an intervening decade, there were reductions from pre-intervention to the 10-year Reunion in both time-to-exhaustion and peak VO_2_ for all groups (Table 1 and Figure 1, 2). However, as compared to 10% peak VO_2_ reductions for the controls and the moderate intensity training group, the two vigorous intensity training groups experienced fitness reductions that were about half as much (∼5%; Figure 1, Panel A). The vigorous intensity group-specific differences appeared to be due, at least in part, to obtaining a higher peak VO_2_ at the end of training (Figure 2, Panel A). In fact, the slopes of the decrease from post training peak VO_2_ to the Reunion time point was steeper for the vigorous intensity exercise groups than for the control group (*P* < 0.0083). The moderate intensity group experienced a steeper slope also, but after controlling for multiple comparisons, the difference from the control group was not significant (*P* = 0.046). If the groups are combined into vigorous intensity and non-vigorous intensity exposures groups, the average fitness reduction of 4.7% for the vigorously trained was significantly different from the reduction of 9.6% for the controls plus moderate intensity groups (*P* < 0.05; data not shown).

For the subset of subjects that had complete VO_2_ at anaerobic threshold data, we also combined groups into vigorous intensity (*n* = 38) and non-vigorous intensity (*n* = 14) exposure groups. Changes in VO_2_ at anaerobic threshold mirrored the changes observed in peak VO_2_. The non-vigorous intensity group displayed a significant 10-year reduction in VO_2_ at anaerobic threshold from baseline to Reunion (16.9 and 15.0 mL/kg/min, respectively; *P* = 0.008). Conversely, the vigorous intensity group experienced a larger increase in VO_2_ at anaerobic threshold post-training and a steeper decreasing slope during the following 10 years compared to the non-vigorous intensity group. Overall, this resulted in an apparent attenuation of decline in submaximal exercise capacity for the vigorous intensity group, as baseline and Reunion VO_2_ at anaerobic threshold values (17.1 and 16.5 mL/kg/min, respectively) were not significantly different (*P* = 0.07). Although not statistically significant between groups (*P* = 0.07), as compared to the 11.1% reduction in VO_2_ at anaerobic threshold for the non-vigorous intensity group, the vigorous intensity group experienced a reduction in VO_2_ at anaerobic threshold that was almost a fourth as much (3.0%) over the 10-year period (Supplementary Figure S1).

Additional analyses showed a significant correlation (*r* = -0.40; *P* = 0.0001) between the subjects’ reported exercise frequency over the last 3 months (questionnaire data taken at the Reunion time point) and the peak VO_2_ reduction over the decade of follow-up (Table 2). That is, in all groups combined, subjects reporting no exercise over the previous 3 months averaged a 12.8% decline in peak VO_2_ over the 10-year follow-up period, whereas those reporting exercising three times per week averaged a 6.1% decline. Finally, those reporting exercising four or more times per week over the last 3 months actually gained 5.5% in peak VO_2_ over the 10-year follow-up period (data not presented). That said, recent exercise explains only 16% of the variance (*r* = 0.40) in the effect on peak VO_2_.

**Table 2:** Correlations between self-reported exercise frequency over the last three months (taken at the STRRIDE Reunion timepoint) and change in cardiometabolic health variables.

Variable	*r*	*P* value	*n*
Peak VO_2_ Change	0.400	0.0001	87
Body Mass Change	-0.300	0.002	100
High Density Lipoprotein Change	0.294	0.003	101
Triglycerides Change	-0.273	0.006	99
Minimal Waist Circumference Change	-0.271	0.007	99
Fasting Insulin Change	-0.103	0.310	100
Fasting Glucose Change	-0.050	0.620	99
Mean Arterial Pressure Change	0.024	0.810	102


### Minimal Waist Circumference

As shown in Table 1, minimal waist circumference increased only in the controls over 10 years. Both low amount training groups had significantly different changes in waist circumference compared to the control group over the follow-up period (Figure 1, Panel D). Interestingly, in the initial training phase (from pre- to post-intervention) the controls did not change in waist circumference (Figure 2, Panel D). However, all three exercise groups experienced decreases in waist circumference during training. All three exercise training groups benefitted from both a decrease in waist circumference during training and a much slower increase in waist circumference over 10 years. As with peak VO_2_, there was a significant correlation between recent exercise frequency and minimal waist change over this period (*r* = -0.27; *P* = 0.007; Table 2).

### Mean Arterial Blood Pressure (MAP)

Ten years after the intervention, none of the groups experienced an increase in MAP. Perhaps this is because approximately one third of the subjects were on blood pressure medications at the time of the Reunion (they were not permitted at study initiation). Of note, those in the moderate intensity group (-7.7 mmHg; *P* < 0.0001) and the high amount/vigorous intensity group (-3.4 mmHg; *P* < 0.05) had significantly lower blood pressures than prior to the intervention (Table 1). Compared to the other three groups, the moderate intensity group experienced significantly greater reductions in MAP (Figure 1). As reported in Table 2, there was no significant relationship between change in MAP and recent self-reported exercise.

### Fasting Insulin

The greatest reduction in fasting insulin occurred in the moderate intensity group; this decrease was significantly greater than the control group and the high amount/vigorous intensity group. As already noted, the assay techniques for fasting plasma insulin before and after the follow-up period were different. Thus, the apparent changes over time are best understood in comparisons among groups. With that in mind, fasting insulin decreased in all three exercise groups but increased in the controls during the STRRIDE study; ten years later, it appears that two of the exercise groups continued to decrease — especially with moderate intensity exercise — and both the high amount/vigorous intensity group and the control group appeared not to change over the 10 years. There was no significant correlation between change in fasting insulin and recent self-reported exercise (Table 2).

### Fasting Plasma Glucose and Medications

Over the decade of follow-up, fasting glucose levels increased substantially across all groups, which was true whether they exercised frequently during the last three months or not, and after controlling for medications. A total of 62 subjects had fasting glucose concentrations in the impaired (> 100 mg/dL; *n* = 52) or diabetic (> 125 mg/dL; *n* = 10) range. In spite of this large and widespread increase in fasting glucose over the follow-up period, only three subjects were taking medications specifically for glucose control. Approximately half of the subjects (*n* = 51) were taking medications that might affect glucose levels (e.g., beta-blockers, thiazides, dedicated glucose lowering medication). When the change in glucose over the decade was controlled for glucose-affecting medications, the increase in fasting glucose was 11.4 versus 15.3 mg/dL, which was not significantly different between those two groups (*P* = 0.14). Furthermore, there was no significant relationship between change in fasting glucose and recent self-reported exercise (Table 2).

## Discussion

In the STRRIDE Reunion study, we evaluated changes in cardiometabolic health parameters 10 years after completion of the original STRRIDE study relative to assessments taken at the time of original randomization; thus, change scores reflect the original study-related changes plus those persisting over the ensuing 10 years—the so-called “legacy effect.” We observed several group-specific legacy effects from the eight-month exercise training programs. These effects, if verified in future studies, would demonstrate the power and value of exercise training programs of even moderate duration for long-term cardiometabolic health benefits.

Ten years after subjects finished the eight-month exercise training study (STRRIDE), several important cardiometabolic health measures were improved and/or maintained better over 10 years of follow-up depending on group assignment and participation. The strongest and most consistent findings with respect to metabolic health variables were observed with moderate intensity exercise. Moderate intensity exercise resulted in persistent beneficial effects for blood pressure, fasting insulin, and waist circumference — all strong measures of metabolic syndrome. Blood pressure responses persisted even after controlling for blood pressure medication use and were similar for systolic blood pressure and diastolic blood pressure (data not shown). These legacy effects of moderate intensity exercise on metabolic syndrome markers are in contrast to the effects of participating in vigorous intensity exercise of any amount. These observations are consistent with our findings from two of our previous STRRIDE studies, where moderate intensity exercise out-performed vigorous intensity exercise of the same amount for glucose control and metabolic syndrome measures (Houmard et al., 2004; Johnson et al., 2007; Slentz et al., 2016).

### Cardiorespiratory Fitness

The legacy effects specific to moderate intensity training did not extend to cardiorespiratory fitness. Conversely, while there were no significant group differences, both the inactive control and the moderate intensity groups experienced fitness losses approximately twice as great as that of the vigorous intensity groups (peak VO_2Control_ = 12.3%; peak VO_2Moderate_ = 10.5%; peak VO_2LowAmt/V ig_ = 4.8%; peak VO_2HighAmt/V ig_ = 5.8%). When combined into two groups (vigorous intensity exercise training versus non-vigorous or no exercise training), the difference between these two categories was significant; this suggests that eight months of vigorous exercise training may lead to a higher fitness level 10 years later. As previous research has consistently reported loss in aerobic fitness of approximately 10% per decade (Rogers et al., 1990; Loe et al., 2013; Kaminsky et al., 2015), our findings suggest that eight months of vigorous training combined with the apparent legacy effects may attenuate as much as half of this expected loss. Furthermore, in our subgroup analyses of VO_2_ at anaerobic threshold, we observed an even greater maintenance of submaximal aerobic capacity as those who completed vigorous intensity training experienced only a 3.0% reduction in VO_2_ at anaerobic threshold over the 10-year period. In order to address whether this subset of subjects potentially had a differential fitness response compared to those who did not have complete anaerobic threshold data, we compared the average change in peak VO_2_ from baseline to Reunion and found no statistical difference.

Although the groups started with similar peak VO_2_ values, the greatest improvements were seen in the vigorous intensity training groups. Thus, one reason for the lesser decline in the vigorous intensity groups appears to be the greater peak fitness gain achieved during the 8-month training period (demonstrated in Figure 2). This is similar to observations in the Look AHEAD trial where post-intervention increases in glucose over time appeared to be consistent among the group assignments (parallel trajectories); the differences among groups at follow-up appeared to be related to the initial intervention-related improvement in glucose control with a relatively similar post-intervention decrement over time. In addition, there was a significant moderate correlation between change in peak VO_2_ and self-reported exercise frequency over the last 3 months (taken at the Reunion timepoint).

### Waist Circumference

Remarkably, when compared with controls, all three exercise groups experienced a legacy effect for waist circumference — that is, all three exercise groups had reduced waist circumferences. These results reflect the fairly stable maintenance of post-intervention waist circumference in these three exercise groups when compared to the large increase observed in the control group. As with peak VO_2_, there was a significant correlation between reported exercise frequency over the three months just before the Reunion visit and change in minimal waist over the entire follow-up period. Taken together, these data suggest that the observed legacy effect of an eight-month exercise training period is due to both the effect of reducing waist circumference during the STRRIDE intervention period, and the effect of frequent exercise over the more recent timeframe (and, although we were not able to measure this, possibly over the duration of the 10-year follow-up period).

We previously observed that moderate intensity exercise may have a greater metabolic benefit than vigorous exercise (Houmard et al., 2004; Johnson et al., 2007; Slentz et al., 2007, 2009, 2016). If the moderate intensity group chose moderate intensity exercise when they resumed or continued their exercise routine over the last 10 years, this could explain the metabolic benefits (better blood pressure and fasting insulin levels) compared to the vigorous groups 10 years after they finished the study. Similarly, for the vigorous groups, if they chose more vigorous exercise when they resumed or continued their exercise routine over the past 10 years, this may help explain their smaller reductions in peak VO_2_.

Both of these possibilities are supported by recent data published from the Look AHEAD study (Group, 2013). Look AHEAD was a multicenter randomized controlled trial in the United States investigating the effects of intensive lifestyle intervention on cardiovascular morbidity and mortality among patients with type 2 diabetes (Ryan et al., 2003). The intensive lifestyle intervention included both group and individual sessions that promoted a weight loss goal of at least 7% through decreased caloric intake and increased physical activity. The control group received diabetes support and education in group-based settings. In their article on the cardiovascular effects of intensive lifestyle intervention in type 2 diabetes, each of the four variables studied over 10 years — body weight, physical fitness, waist circumference and glycated hemoglobin —robustly improved in the lifestyle group compared to the diabetes support and education group during the first year of the intervention (Group, 2013). While these large differences were quickly reduced, becoming much less during the next few years, there were still differences for each of these variables with the lifestyle group still significantly better compared to the diabetes support and education group at each time point of follow-up. This suggests a benefit of a much-improved “baseline” which persisted for several years. In another paper from the Look AHEAD study group, at least part of this benefit was due to greater levels of continued physical activity (Unick et al., 2016). They observed that when measured objectively, the intensive lifestyle group engaged in significantly more moderate-to-vigorous physical activity than the diabetes support and education group over a four-year period. These data suggest that an intensive intervention can provide long-lasting effects both by contributing to better immediate post-intervention health as well as long-term maintenance of healthy behaviors.

There are a number of limitations to the current study. In STRRIDE, all subjects in the control group were offered exercise training after the control period. Those deciding to not join an exercise group constituted the control group; thus, the control group may be biased toward persons maintaining lifelong inactivity. Similarly, we did not invite persons that “dropped out” of any arm back for the Reunion study. In a future study, to increase the generalizability of the study and allow us to make comparisons between those who successfully completed the exercise training and those who did not, we plan to invite back subjects who dropped out of either the control group or any of the exercise groups. This would allow us to evaluate whether or not completing a significant exercise training program can have an effect 10 years later on overall health.

Our findings indicate that a relatively modest period of regular exercise training creates legacy effects over the ensuing 10 years; these findings are consistent with those of other behavioral interventions. The patterns observed indicate that as compared to those without a defined exercise training exposure, vigorous exercise training offers benefits for maintaining aerobic fitness; moderate intensity exercise training produces sustained legacy effects on metabolic parameters; and any exercise training helps maintain body weight and waist circumferences better than continued inactivity. The importance of these findings is hard to overestimate from both individual and global health viewpoints. If a single, prolonged 8-month exercise training study can lead to behavior changes that continue for 10 years — with the attendant health benefits — this would have potentially sizeable and widespread beneficial effects. The observed responses may not be purely behavioral; they may also be partly mediated by some contributions from epigenetic or other biological effects. These possibilities warrant further study. Irrespective of the mechanisms, the observed 10-year consequences of an 8-month aerobic exercise intervention include better weight control, greater retained aerobic fitness for vigorously exercise-trained individuals, and improved metabolic profiles for moderate intensity-trained individuals. In conclusion, as both the individual and public health implications are substantial, conducting more studies designed to specifically test for the possible health and/or fitness legacy effects of a single prolonged exercise training program is imperative.

## Ethics Statement

This study was carried out in accordance with the recommendations of the Duke University Institutional Review Board with written informed consent from all subjects. All subjects gave written informed consent in accordance with the Declaration of Helsinki. The protocol was approved by the Duke University Institutional Review Board.

## Author Contributions

JJ and WK designed the research. JJ performed data collection. JJ, CS, LR, and WK analyzed data and interpreted results. JJ, CS, LR, KH, and WK participated in writing and revising the manuscript. All authors approved the final version of the manuscript.

## Conflict of Interest Statement

The authors declare that the research was conducted in the absence of any commercial or financial relationships that could be construed as a potential conflict of interest.

## Supplementary Material

FIGURE S1 Percent change in peak VO_2_ and VO_2_ at anaerobic threshold from pre-STRRIDE to STRRIDE Reunion (10 years and 8 months later) for subgroup of participants with complete anaerobic threshold data. Participants were categorized into non-vigorous and vigorous intensity exposure groups. AT, anaerobic threshold.
