# Supplementary figures and images for: Ten-Year Legacy Effects of Three Eight-Month Exercise Training Programs on Cardiometabolic Health Parameters
Source: Front Physiol. 2019 Apr 16;10:452. doi: 10.3389/fphys.2019.00452 (PMC6476955; doi:10.3389/fphys.2019.00452)

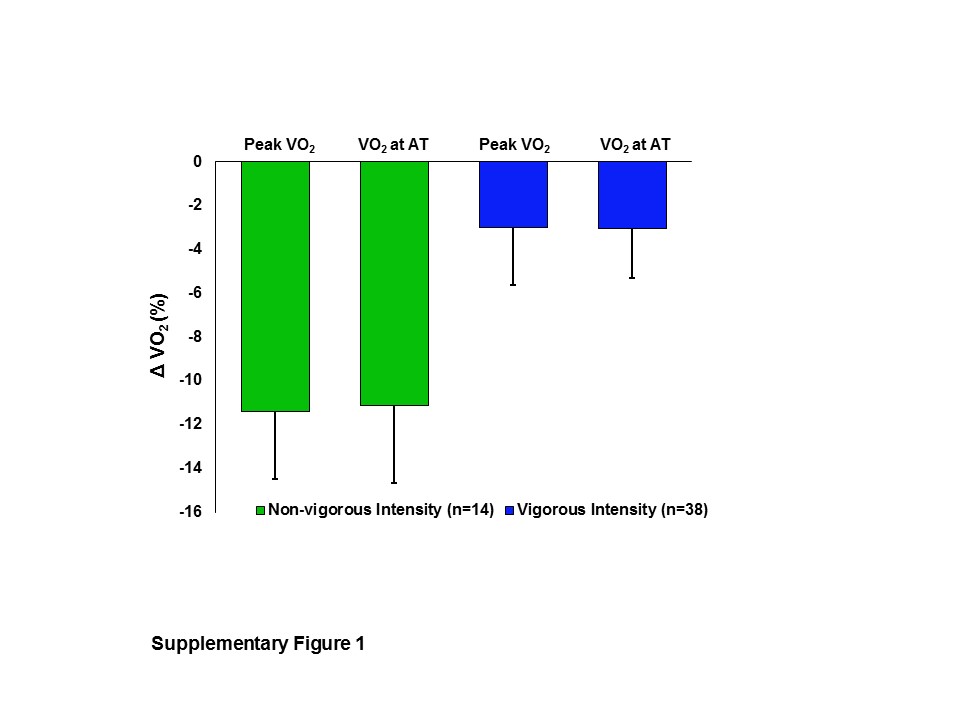

Supplement: FIGURE S1 — Percent change in peak VO2 and VO2 at anaerobic threshold from pre-STRRIDE to STRRIDE Reunion (10 years and 8 months later) for subgroup of participants with complete anaerobic threshold data. Participants were categorized into non-vigorous and vigorous intensity exposure groups. AT, anaerobic threshold. [file Image_1.JPEG]
